# The Pharmacopsychometric Triangle to Illustrate the Effectiveness of T-PEMF Concomitant with Antidepressants in Treatment Resistant Patients: A Double-Blind, Randomised, Sham-Controlled Trial Revisited with Focus on the Patient-Reported Outcomes

**DOI:** 10.1155/2011/806298

**Published:** 2011-06-07

**Authors:** P. Bech, M. Gefke, M. Lunde, L. Lauritzen, K. Martiny

**Affiliations:** ^1^Psychiatric Research Unit, Frederiksborg General Hospital, 3400 Hillerød, Denmark; ^2^Mental Health Research Unit, Psychiatric Centre, 2100 Copenhagen Ø, Denmark

## Abstract

*Background*. Our T-PEMF trial has been revisited with focus on the pharmacopsychometric triangle in which effect size is used when comparing wanted versus unwanted clinical effects and quality of life as outcomes. In this analysis, we have especially focused on the self-reported HAM-D_6_. *Methods*. The antidepressive medication which the patients were resistant to was kept unchanged during the five weeks of active versus sham T-PEMF. *Results*. In total 21, patients received active T-PEMF, and 19 patients received sham T-PEMF. The effect size was 1.02 and 0.90, respectively, on HAM-D_6_ and HAM-D_6_-S. Concerning side effects, the active T-PEMF reduced the baseline score on concentration problems with an effect size of 0.44 while inducing more autonomic symptoms than sham T-PEMF with an effect size of −0.41. The advantage of active over sham T-PEMF obtained an effect size of 0.48. *Conclusion*. Active T-PEMF was found superior to sham T-PEMF within the pharmacopsychometric triangle with a clinically significant effect size level above 0.40.

## 1. Introduction

In our trials on the concomitant use of repetitive transcranial magnetic stimulation (rTMS) or transcranial pulsed electromagnetic fields (T-PEMF) and antidepressants in patients with treatment-resistant depression [1–3], we have focussed on the clinician-administrated depression scales, for example, the Hamilton depression scale (HAM-D_17_) or the Montgomery-Åsberg depression scale (MADRS).

Over the last decade-patient-reported depression scales have been found important as a supplemental assessment of outcome in trials of antidepressants [4–6]. Moreover, the pharmacopsychometric triangle has been introduced to give an overall clinical outcome profile of an intervention [7, 8], taking into account the wanted antidepressive effect, the unwanted side effects, and the patients' own balanced evaluation of health-related quality of life. The pharmacopsychometric triangle is scientifically connected to effect size statistics [9, 10], as it allows us to evaluate diverse outcome scales (wanted versus unwanted effect as well as subjective quality of life) by reference to an integrated unit, namely, the standard deviation of the improvement scores [7].

In our T-PEMF study [2], we included the patient-reported HAM-D_6_-S [5] just after this trial was started. Thus, 40 out of the 50 original patients included in the analysis reported here have completed the patient-administrated HAM-D_6_-S.

The objective of this study was to revisit the T-PEMF study by Martiny et al. [2] with focus on the 40 patients who had completed the HAM-D_6_-S and to compare the results, on the one hand, with the clinician-administrated HAM-D_6_ for evaluation of the pure antidepressive outcome and, on the other hand, to use the pharmacopsychometric triangle and the related effect size statistics, as the WHO-5 well-being index [10] was included as a measure of quality of life. As for side effects evaluation, the UKU scale was reanalysed [11]. 

## 2. Methods

### 2.1. Ethics

The study [2] was approved by the Committee on Biomedical Research Ethics and the Danish Central Data Register. Patients were given information as requested by the Biomedical Research Ethics, and the patients all signed an informed consent form.

The study was performed according to the Declaration of Helsinki and the European Union Directive of Good Clinical Practice [12] and was monitored by an external contract company (Encorium, Denmark). 

### 2.2. Patients

In total, 40 patients (12 males and 28 females) with treatment resistant depression were included in the T-PEMF revisited study. In total, 21 of these patients received active T-PEMF, and 19 patients received sham PEMF. The patients were diagnosed as having major depression according to DSM-IV and treatment resistance with a required score of 3 or more on the Sackeim scale [13]. 

### 2.3. Psychopharmacological Treatment

The psychopharmacological treatment for depression (antidepressants and mood stabilizers), to which the patients had developed resistance, was kept unchanged during the 4 weeks preceding baseline and was maintained at the same dose level throughout the study. However, zopiclone was permitted to treat emergent sleep problems. Otherwise, no other change in ongoing psychopharmacological treatment was allowed.

Most of the patients received more than one antidepressant (escitalopram, other SSRIs, and dual active antidepressants (venlafaxine, duloxetine, and mirtazapine) and tricyclics. Moreover, 7.5% received lithium and 20% received anticonvulsants as mood-stabilising drugs. 

### 2.4. Psychometrics

#### 2.4.1. Diagnosis

The DSM-IV diagnosis of major depression was assessed by use of the MINI International Neuropsychiatric Interview (MINI) [14]. 

#### 2.4.2. Clinician Administered Depression Rating Scales

The Hamilton Depression Scale (HAM-D) was used in the HAM-D_17_ version accepted by Hamilton [15]. The HAM-D_6_ version includes the six depression items found clinically valid [16] and psychometrically valid [17] when measuring the pure antidepressive effect of a drug [18]. These six items are depressed mood, guilt feelings, work and interests, psychomotor retardation, psychic anxiety, and general somatic (tiredness and/or pains). The patient-administrated HAM-D_6_-S is the pencil-and-paper version released by Bech et al. [5]. 

The theoretical score range of the HAM-D_17_ goes from 0 to 52, whereas the theoretical score range of HAM-D_6_ goes from 0 to 22.

The melancholia scale (MES) is an eleven item version based on the HAM-D_6_. It has also been found both clinically and psychometrically valid when measuring the severity of depressive states [19, 20]. 

The theoretical score range of MES goes from 0 to 44. 

#### 2.4.3. Patient-Rated Depression Scales

Both the self-rated version of HAM-D_6_ (see above) and the major depression inventory (MDI) [21] were used.

The MDI covers the items within the DSM-IV concept of major depression, and its total score has been found psychometrically valid. The theoretical score range of MDI goes from 0 to 50. 

#### 2.4.4. Clinician Administered Side Effect Scale

From the UKU (Udvalg for Kliniske Undersøgelser, Committee for Clinical Trials) [11], the 24 items for antidepressants [22] were included. These 24 items consist of 10 items capturing psychic side effects (e.g., concentration disturbances or sleep problems), 2 items covering neurological side effects (e.g., tremor), 8 items covering autonomic anxiety symptoms (e.g., nausea, constipation, diarrhoea, and increased sweating) and 6 items covering such symptoms as weight gain, headache, and sexual dysfunction.

The theoretical score range of the UKU-24 in its original version goes from 0 to 72, as each item is scored on a Likert scale from 0 (not present) to 3 (severely to extremely present) [22]. However, in this study, the patients were expected to be characterized by having a “tolerable” side effects profile at the time of randomization (baseline), implying that the score on each UKU-24 item goes from 0 (not present) to 1 (clearly present), but not interfering with daily functioning. The theoretical score range is consequently from 0 to 24. 

#### 2.4.5. Quality of Life Scale

The WHO-five well-being index was used. This scale contains five items covering psychological well-being. The total score range goes from 0 (lowest level of well-being) to 100 (highest level of well-being) [10]. 

#### 2.4.6. T-PEMF Procedure

The T-PEMF procedure has been described elsewhere in detail [2]. In brief, the T-PEMF delivery system consists of a 220 V pulse generator, which provides pulses to the applicator constructed as a treatment helmet. The dimensions of the Re5 T-PEMF generator are (width × height × depth) 2.8 × 1.6 × 9.2 inches. The pulses provided by the generator to the coils in the helmet alternate between +50 and −50 V. The treatment helmet incorporates, on the inner side, 2 coils in the anterior and posterior temporal region on both sides and 1 coil in the upper parietal region on both sides and 1 coil in the centre of the lower occipital region. Thus, in total, 7 coils are connected in parallel with the pulse generator. The Re5 T-PEMF pulse generator powers the helmet with alternating bipolar square pulses each lasting 3 ms and interspersed by a 12 ms pause, each pulse sequence thus lasting 18 ms, corresponding to a pulse frequency of 55 Hz. The rapid change of the current in the coils from the pulse generator creates an alternating magnetic field capable of inducing electrical fields in tissue with an intensity of 2.5 mV/cm at 2 cms from the individual coil [23]. 

In comparison, the depolarization (35 mV) required for induction of an action potential by opening of Na^+^ channels over a 10 nm wide plasma membrane is in the order of 3.5·10^6^ V/m or 3.5·10^7^ mV/cm and is, therefore, many orders of magnitude larger than the electrical field imposed by the T-PEMF treatment (2.5 mV/cm). In addition, the fields in the human cortex induced by the T-PEMF system are very much lower than those applied by rTMS equipment, which uses stimuli approaching neuronal firing level.

Patients came for daily sessions on all weekdays (Monday–Friday) for 5 weeks at the two including centres. Treatment was supervised to secure an accurate activation of the generator and compliance with the 30 minute session. 

#### 2.4.7. Statistical Analysis

Effect size was used to compare active T-PEMF with sham T-PEMF. The standardized effect size was calculated using the last observation carried forward (LOCF) approach for missing values. The standardized effect size is a descriptive statistic and is defined as the difference in mean change score from baseline to study endpoint between active T-PEMF and sham T-PEMF divided by the pooled standard deviation [24]. When the difference in mean score from baseline to endpoint is positive, the effect size is positive, indicating in the HAM-D_6_ score for example that active PEMF is superior to sham PEMF as a decrease in the symptom score reflects an antidepressive effect. For the WHO-5 well-being index, in which an increase in total score reflects greater well-being, the difference in mean score from baseline to endpoint is negative if active PEMF is superior to sham PEMF, resulting in a negative effect size.

Cohen [9] considered an effect size of 0.50 to be of clinical significance, but in placebo-controlled clinical trials of antidepressants in nontreatment resistant depressed patients, an effect size of 0.40 or higher is considered clinically significant [18] which is in agreement with Baer and Blais [25]. 

## 3. Results

Out of the 40 patients included in the study, five patients dropped out before the planned duration of the trial period. Four of these patients received active T-PEMF, and one received sham T-PEMF. Among the patients receiving active T-PEMF, one patient dropped out due to change in the antidepressive medication, one patient due to lack of improvement, one patient did not wish to continue, and one patient stopped due to vacation. The patient dropping out in the sham T-PEMF group did so due to change in antidepressive medication.

Table 1 shows the baseline characteristics of all 40 patients together and when subdivided into the 21 patients receiving active T-PEMF and the 19 patients receiving sham T-PEMF. No statistically significant differences were observed apart from age, as the active T-PEMF group of patients was older than the sham group (58.2 versus 49.5 years of age (*P* ≤ .05)).

**Figure 1:**
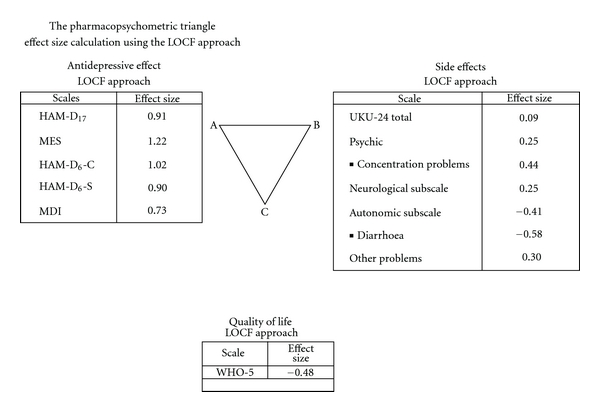


Figure 1 shows the pharmacopsychometric triangle in which the pure antidepressive effect is based on the HAM-D_17_ and MES and on the clinician- versus patient-administrated HAM-D_6_-S as well as on the self-rated MDI. In general, the clinician-administrated scales (MES, HAM-D_17_, and HAM-D_6_) obtained higher effect sizes than the patient-administrated scales (HAM-D_6_-S and MDI), but all the effect sizes were above 0.50.  The side effects as measured by the clinician-administrated UKU-24 showed an effect size of 0.09.

As expected, the patients typically had at randomisation a score range on the individual UKU items from 0 (not present) to 1 (clearly present but not interfering with daily functioning). However, for the psychic UKU side-effect items such as “concentration difficulties”, “tension/inner unrest”, and “increased dream activity”, the score range lay between 0 and 2 (clearly present but without influencing patient's daily life to any marked degree). 

Table 2 shows the total scores on the UKU-24 at baseline (randomisation) and each week during the five weeks of treatment. At baseline, the total score was approximately 12, and it decreased during the five weeks of therapy in both groups of treatment with, however, the numerically greatest decrease in the active T-PEMF group of patients. Therefore, the effect size of 0.09 is positive. In Figure 1, we have also indicated the effect size for the UKU subscales of psychic, neurological, autonomic, and other symptoms. For the UKU subscale of psychic problems, the effect size was 0.25. Within these symptoms the item of concentration disturbances obtained an effect size above 0.40, indicating that these symptoms achieved a better improvement on active than on sham T-PEMF. As regards the UKU subscale of autonomic symptoms, the effect size was −0.41, indicating that these symptoms reached a better improvement on sham T-PEMF than on active T-PEMF. Within the autonomic symptoms, the item of diarrhoea obtained an effect size of −0.58. 

On the MES item of concentration disturbances the effect size was 0.50.

With regard to the WHO-5 well-being index, higher scores reflect higher quality of life explaining the negative effect size sign of −0.48 (Figure 1) to illustrate the advantage of active T-PEMF over sham T-PEMF.

Table 3 shows the week-to-week scores on the HAM-D_6_ clinician version when compared to the patient-administrated HAM-D_6_-S. In general, the standard deviations were numerically higher in the patient-administrated version of HAM-D_6_ compared to the corresponding scores on the clinician-administrated version. On the clinician HAM-D_6,_ the difference between active versus sham T-PEMF was statistically significant as early as after one week of therapy (*P* ≤ .05). However, on the patient-administrated HAM-D_6_-S, the level of statistical significance after 3 weeks of therapy was greater (*P* ≤ .01) than on the corresponding clinician HAM-D_6_ (*P* ≤ .05).

On the MDI (data not shown) the difference between active and sham T-PEMF was first seen after 4 weeks of therapy at a level of significance of *P* ≤ .05.

Table 4 shows the LOCF analysis for the HAM-D_17,_ and Table 5 shows the LOCF analysis for MES. For both scales, the difference between active and sham T-PEMF was statistically significant already after the first week of treatment. 

## 4. Discussion

As in our previous study [8], the pharmacopsychometric triangle was found to have a high degree of communicative validity. The effect size statistics when comparing active T-PEMF with sham T-PEMF clearly indicated the superiority of the active T-PEMF in terms of antidepressive effect and the patients' self-reported quality of life.

According to the most recent updating of the standardization of Cohen's effect size statistics [25] in relation to clinically significant effects, the interval between 0.00 and 0.19 refers to no effect; 0.20 and 0.39 refers to a small effect; the interval between 0.40 and 0.69 refers to a medium effect; the level of 0.70 or higher refers to a large effect. Our pharmacopsychometric triangle which is linked to Cohen's effect size statistics showed a large clinically significant effect with regard to antidepressive effect, both on the clinician-rated outcome scale and on the patient-rated scales.

The self-rating HAM-D_6_-S obtained an effect size in favour of active T-PEMF of 0.90, that is, as high as for HAM-D_17_.

As regards the side effects of the combination of antidepressive maintenance therapy and T-PEMF, the baseline UKU scores decreased in both active and sham T-PEMF group of patients. The decrease in the UKU symptom of concentration disturbances was much higher in the active T-PEMF-treated patients resulting in an effect size of 0.44, that is, medium effect clinically. This was parallel to the MES item of concentration disturbances with an effect size of 0.50. In other words, concentration disturbances seem to be an important feature of the therapy-resistant patients; this is in agreement with our principal component analysis of the Montgomery-Åsberg depression scale (MADRS) and the MES [3]. 

On the other hand, the active T-PEMF had less improvement on the UKU subscale of autonomic symptoms compared to sham T-PEMF. It was especially the item of diarrhoea that resulted in this difference, with an effect size of −0.58. However, the effect size of 0.09 on the total UKU-24 indicated no clinically significant difference between active and sham T-PEMF. In total, five patients dropped out of the study: four patients during active T-PEMF and one patient during sham T-PEMF. In other words, only 12.5% of the initial 40 patients at baseline dropped out.

In the patient-reported WHO-5 well-being index quality of life assessment, on which high scores signify better well-being, the effect size level was −0.48, indicating an advantage of active T-PEMF over sham T-PEMF of a medium clinically significant magnitude. As discussed elsewhere in trials of antidepressants, the effect on quality-of-life measurements is often of clinical significance after a treatment period longer than 5 weeks [26].

The self-reported HAM-D_6_-S was in this study found to be as sensitive as the clinician-administrated HAM-D_6_. In our previous psychometric validation study of HAM-D_6_-S, the use of item response theory models demonstrated that the total score was sufficient [6].

In conclusion, this reanalysis of the T-PEMF study [2], focussing on the pharmacopsychometric triangle with its use of effect size statistics and incorporating the self-reported HAM-D_6_-S, has confirmed the superiority of active T-PEMF over sham T-PEMF in patients maintaining the antidepressive medication they had been resistant to. In the previous T-PEMF report, the mixed model (the random-effects regression model) was used for the intent-to-treat approach [2], but in this revisited study, we have used a conservative intent-to-treat approach by carrying forward the last observation (LOCF). Even within this conservative approach, we were able to confirm the superiority of active T-PEMF over sham T-PEMF. 

## Figures and Tables

**Table 1:** Baseline characteristics.

Baseline characteristics	T-PEMF		
	Active	Sham	All
	(*N* = 21)	(*N* = 19)	(*N* = 40)
Age, mean (sd) [range, years]	58.2 (12.9)* [35–85]	49.5 (11.9) [24–70]	54.0 (13.0) [24–85]
Duration of episodes (month)mean (sd)	2.2 (2.5)	3.2 (5.0)	2.7 (4.1)
% females	66.7%	63.2%	65.0%

*P ≤ .05.

**Table 2:** Side effects as total scores on UKU-24. LOCF analysis.

UKU-24	Active T-PEMF	Sham T-PEMF
	(*N* = 21)	(*N* = 19)
Baseline	12.3 (2.6)	13.5 (2.3)
week 1	11.4 (3.4)	12.2 (2.7)
week 2	10.0 (3.4)	11.2 (2.9)
week 3	9.7 (3.1)	11.1 (2.4)
week 4	9.0 (3.0)	9.8 (2.1)
week 5	8.6 (3.8)	10.2 (2.3)

**Table 3:** Comparison of clinician-rated and patient-rated HAM-D_6_. LOCF analysis.

HAM-D_6_	Clinician		Patient	
	Active T-PEMF	Sham T-PEMF	Active T-PEMF	Sham T-PEMF
	(*N* = 21)	(*N* = 19)	(*N* = 21)	(*N* = 19)
Baseline	12.5 (2.1)	12.5 (2.0)	13.9 (2.2)	13.8 (2.5)
week 1	10.6 (2.9)*	11.7 (2.1)	10.9 (3.8)	11.2 (3.3)
week 2	9.7 (2.2)*	10.6 (2.8)	9.3 (3.0)*	11.5 (4.4)
week 3	8.8 (2.3)*	10.6 (3.1)	8.6 (3.4)**	11.9 (4.0)
week 4	7.9 (3.0)*	10.8 (2.8)	7.8 (3.3)**	12.0 (4.0)
week 5	6.9 (3.7)*	10.5 (3.2)	7.0 (4.5)**	10.8 (3.7)

*P ≤ .05.

**P ≤ .01.

**Table 4:** The LOCF analysis with HAM-D_17_.

HAM-D_17_	Active T-PEMF	Sham T-PEMF
	(*N* = 21)	(*N* = 19)
Baseline	21.29 (4.0)	21.62 (3.6)
week 1	17.33 (5.1)*	19.58 (3.3)
week 2	15.05 (3.2)*	17.63 (4.9)
week 3	14.10 (4.5)**	17.68 (4.9)
week 4	12.67 (5.6)**	17.79 (4.0)
week 5	11.38 (6.5)**	16.68 (4.4)

*P ≤ .05.

**P ≤ .01.

**Table 5:** The LOCF analysis with MES.

HAM-D_17_	Active T-PEMF	Sham T-PEMF
	(*N* = 21)	(*N* = 19)
Baseline	21.62 (3.1)	21.21 (2.7)
week 1	17.90 (5.0)**	20.00 (3.5)
week 2	16.33 (3.2)*	18.68 (4.2)
week 3	14.95 (4.0)**	18.74 (4.5)
week 4	13.05 (5.1)**	18.16 (4.8)
week 5	11.90 (5.9)**	17.79 (4.7)

*P ≤ .05.

**P ≤ .01.
